# Reproductive outcomes after hysteroscopic metroplasty for women with dysmorphic uterus and recurrent implantation failure

**Published:** 2018-06

**Authors:** J Ferro, E Labarta, C Sanz, P Montoya, J Remohi

**Affiliations:** Director of Surgery of Instituito Valenciano de Infertilidad “IVI”, Plaza de la Policía Local, 3. 46015. Valencia, Spain; Especialist in Reproductive Medicine of Instituito Valenciano de Infertilidad “IVI”; Fellows in Reproductive Medicine of Instituito Valenciano de Infertilidad “IVI”; Director of Instituto Valenciano de Infertilidad “IVI

**Keywords:** dysmorphic uterus, hysteroscopy, metroplasty, pregnancy rate, reproductive outcome, RIF, recurrent implantation failure

## Abstract

**Background:**

The aim of this study was to assess the reproductive outcomes of women with recurrent implantation failure (RIF) after hysteroscopic metroplasty for dysmorphic uteri.

**Methods:**

This retrospective observational study included 190 women with a diagnosis of RIF. These patients were eligible for hysteroscopic metroplasty for dysmorphic uteri, including T-shaped uteri, between January 2008 and September 2015 at the Instituto Valenciano de Infertilidad (IVI) in Valencia, Spain.

**Results:**

The total clinical pregnancy rate, the live birth rate, and the abortion rate were 80.0% (152/190), 77.9% (147/190) and 8.9%, respectively. At 12 months, the clinical pregnancy rate was 76.3% (145/190) and at 6 months 50.5% (96/190). After the metroplasty, approximately 76% of all gravidities, were achieved during the first 12 months of follow-up. Within the first IVF cycle, pregnancy and live birth rates were 77.8% and 86.1%, respectively. The mean time to pregnancy was 6.5 months.

**Conclusion:**

This study demonstrates that hysteroscopic metroplasty improves pregnancy and live birth rates for women with a history of recurrent implantation failure and dysmorphic uterus. However, conclusions must be taken carefully as this is an observational study. A prospective, randomized and controlled study is necessary to support these results.

## Introduction

Despite the scientific advances in reproductive medicine during the last years, recurrent implantation failure (RIF) remains a challenging and extremely disappointing problem for the clinicians and patients (Potdar et al., 2012). It is defined as failure to achieve a clinical pregnancy after the transfer of at least four good-quality embryos (minimum of three fresh or frozen cycles) (Coughlan et al., 2014). Other authors define RIF as the impossibility to achieve conception after 2 to 6 IVF cycles (with high-quality embryos) (Dalton et al., 2006). The successful outcome of a pregnancy depend on several factors, among which embryo quality and intrauterine environment play major roles. Both factors allow for the achievement and continuation of gestation (Raju et al., 2006; Ghahiry et al., 2014). Attention has recently focused on the anatomical integrity of the uterine cavity, as a prerequisite for a receptive endometrium (Saravelos et al., 2008; Di Spiezo Sardo et al., 2015). Indeed, several benign uterine conditions, including Müllerian anomalies, may explain low pregnancy rates in assisted reproductive technology (ART) (Urman et al., 2005; Bozdag et al., 2008).

The uterus is formed from the paramesonephric ducts, called Müllerian ducts, at around 8–16 weeks of fetal life. This process comprises three stages: (i) the organogenesis, or the development of the two Müllerian ducts; (ii) lateral fusion, in which the lower part of Müllerian ducts merges and forms the upper part of the vagina, cervix, and uterus. Lastly, (ii) the reabsorption of the septum that is formed after the fusion of Müllerian ducts. This reabsorption begins at 9 weeks leaving a single central cavity and cervical canal (Saravelos et al., 2008). Hence, uterine malformations may result from disturbance in the developmental process of the Müllerian ducts or from fusion failure (Ribeiro et al., 2009).

Recurrent miscarriage is diagnosed in women with congenital uterine anomalies (Serensen, 1988; Kupesic, 2001). It is not easy to determine the actual incidence of uterine malformations in the general population because most affected women do not present reproductive problems (Valle and Ekpo, 2013). For some authors, an arcuate uterus is the most common in both, the general and recurrent miscarriage populations. For others, a septate uterus is the most common on those with infertility (Raga et al., 1997; Saravelos et al., 2008). However, according to an earlier study of Saravelos [Saravelos et al., 2008], the prevalence of congenital Müllerian anomalies in the general population would be around 6.7%, whereas in the infertile population this percentage attains 7.3% and up-to 16.7% in women facing recurrent miscarriages (Saravelos et al., 2008).

In this instance, different approaches to classify and manage uterine malformations have been proposed (Valle and Ekpo, 2013; Ludwin and Ludwin, 2015). The European Society of Human Reproduction and Embryology (ESHRE) and the European Society for Gynaecological Endoscopy (ESGE) created the Congenital Uterine Anomalies Group (CONUTA) stating a new classification system for uterine anomalies based on a simple anatomical classification (Grimbizis et al., 2013; Sardo et al., 2015). A Class U1 (dysmorphic uterus) integrates all uteri with a normal outline but with an abnormal uterine cavity shape (excludes septa). This Class U1 can be subdivided into three categories: (i) Class U1a (T-shaped uterus), described by thickened lateral walls with a correlation 2/3 uterine corpus and 1/3 cervix that narrow the uterine cavity; (ii) Class U1b (uterus infantilis), also characterized by a narrow uterine cavity without lateral wall thickening and an inverse correlation (1/3 uterine body and 2/3 cervix); and (iii) Class U1c (others), containing all minor malformations of the uterine cavity (Grimbizis et al., 2013).

**Figure 1:**
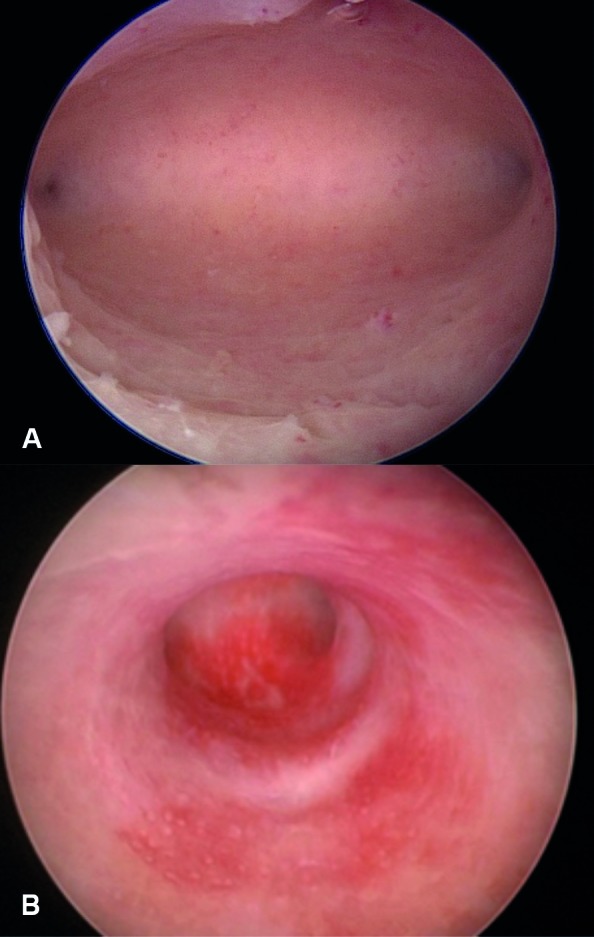
A- Normal cavity B- T shape uterus.

Also, the American Society for Reproductive Medicine (ASRM) proposed its classification in 1988. The later has been widely used and includes T-shaped uteri into the Class VII, relating its cause to diethylstilbestrol-related (DES) exposure (The American Fertility Society, 1988; Valle and Sciarra, 1988; Saravelos et al., 2008; Ludwin and Ludwin, 2015).

It is assumed that malformed uteri-derived infertility is caused by an altered endometrial lining responsible for low implantation rates (Chan et al., 2011). Several studies have shown that metroplasty (using microscissors, electrosurgery or laser) improves the reproductive outcome of infertile women, independently from the subtype of the malformation (Fedele et al., 1993; Pabaçcu and Gomel, 2004; Zlopasa et al., 2007; Bakas et al., 2012). Therefore, compared to abdominal approaches, metroplasy is nowadays considered the first therapeutic option to treat dysmorphic uteri. Some authors recommend it in patients with recurrent pregnancy loss and premature labor to improve obstetric outcomes, as besides considered safe to perform, it offers advantages such as shorter operating time and hospitalization (Pabaçcu and Gomel, 2004; Bakas et al., 2012). However, it is not yet entirely clear whether hysteroscopic metroplasty used in patients with RIF and dysmorphic uteri may improve their reproductive outcomes.

Thus, this retrospective, observational study evaluates the reproductive outcomes of women with recurrent implantation failure after hysteroscopic metroplasty. For this, we adjusted the morphology of the uterine cavity for dysmorphic uteri.

## Materials and methods

### Patients

One hundred ninety women, aged between 25 – 48 years old (mean, 36.8), presenting recurrent implantation failure (defined for this study as ≥ 5 pre- embryos transferred), and posterior hysteroscopic diagnosis of a dysmorphic uterus [including T-shaped uteri] were consecutively analyzed in this study. The duration of infertility in the patient cohort ranged from 1 to 15 years (mean, 4.3 years).

The primary objective of the investigation was the assessment of the reproductive outcome after hysteroscopic metroplasty concerning the live-birth rate. For this, firstly, a complete history, thorough clinical examination and an exhaustive infertility investigation was completed. The later included semen analysis, endocrine evaluation, ovulation assessment and diagnostic imaging. Transvaginal ultrasound was performed to assess the pelvic, uterine and ovarian morphology and anatomy. The initial diagnosis of the dysmorphic uterus was made by hysteroscopy. Informed consent from all patients, regarding the present study and metroplasty, was obtained before every procedure. In all cases, the surgical procedure was performed under general anesthesia, during the early proliferative phase of the cycle and by the same surgeon (J.F.). For consistency, this study also documents the implantation rate, pregnancy rate, abortion and intrauterine death rate.

Approval from the Institutional Research Ethical Committee and the review board was obtained before the initiation of the study. This retrospective observational study took place between January 2008 and September 2015 at the Instituto Valenciano de Infertilidad (IVI) in Valencia, Spain.

### Metroplasty

Metroplasty was performed with a 4.2-mm hysteroscope and a 30° telescope. An additional external sheath, for continuous flow, and a 5-French work channel, for the use of both, scissors and bipolar electrodes was mounted (Karl Storz, Tuttlingen, Germany). The uterine cavity was distended with normal saline solution (0.9%) at an inflow pressure of 70–100 mm Hg. To guarantee minimal systemic absorption during surgery and early recognition of excess fluid deficit, inflow and outflow fluid volumes were measured (Worldwide AAMIG, 2013). There were no patients with fluid deficit of more than 2000 ml.

The endocervical canal was inspected at the beginning of the procedure. Once inside the uterine cavity, a systematic examination was performed by a general evaluation of the uterine cavity starting from the isthmic region. Then, an assessment of the uterine fundus, the side walls, the anterior and posterior walls, horns and tubal ostia was performed.

If a dysmorphic cavity was diagnosed, hysteroscopic metroplasty was performed, with microscissors and a high-frequency bipolar electrode [with further selective coagulation of bleeding vessels] in the same session. The surgical procedure consisted in performing an incision (or straight cuts) at the level of the prominent lateral myometrial walls and to widen it after the incision. Nine and three hours from the isthmic region and in the direction towards the tubal orifices were used as guides. The operation was considered complete when the tubal orifices were seen from the isthmic area of the body, the hysteroscope could be moved freely from one tubal ostium to the other, and a normal uterine cavity of triangular aspect was obtained. Many of these uteri also had some degree of a fundal notch. These required remodeling with scissors. Autocross-linked hyaluronic acid (Hyalobarrier©, Fidia Advanced Biopolymers SRL, Padova, Italy) was introduced in the uterine cavity after surgery as a mechanical barrier between the uterine walls thus preventing the formation of adhesions (Ferro and Montoya, 2016).

**Figure 2:**
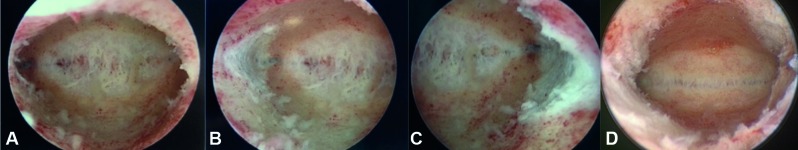
Metroplasty: A — Cut in the prominent fundus of cavity in uterus T shape, like fundus of arcuate cavity. B — Cut in lateral right wall of the uterine cavity. C — Cut in lateral left wall of the uterine cavity.D — Panoramic view of metroplasty.

### Postoperative Measures

In the same day, four to six hours after the procedure, treated patients were discharged. All patients received one dose of azithromycin orally (1 gr), analgesia with paracetamol and hormonal therapy (oestradiol valerate - levonorgestrel) for twenty- one days. In one hundred eighty patients (94.7%), a second-look hysteroscopy was performed after the first deprivation bleeding. Women were evaluated on the first postoperative day and returned for a follow-up visit approximately two months later, for assessment of surgical outcomes.

### Statistics

Data was obtained from SIVIS software (SAP systems, SIVIS Karlsruhe, Germany), and a database was created in Excel (Microsoft® Excel for Mac Ver. 15.32. 2017. Redmond, WA). Statistical analysis was carried out with a statistical software program (SPSS 17.0.1 for Windows; SPSS, Chicago, IL).

## Results

The study included 190 women. The mean age and mean time of infertility were 36.8 years old ± 11.5 and 4.3 years (1 – 15 years), respectively. Metroplasty was performed in women with RIF and a dysmorphic uterus. No cases of uterine perforation were seen, and no case of postoperative fever was recorded. There were no complications related to the fluid deficit during the procedure, and neither to blood loss. If considered appropriate, hyaluronic acid (Hyalobarrier®) was used in 40.5% (77/190) of patients immediately after surgery. All patients were discharged the same day. The total clinical pregnancy rate, the live birth rate, and the abortion rate were 80.0% (152/190), 77.3% (143/190) and 11.1% (17/152), respectively (Table I). Interestingly, at 12 months, the clinical pregnancy rate was 76.3% (145/190) and at 6 months 50.5% (96/190). In other words, approximately 76% of total gravidities after the metroplasty were achieved during the first 12 months of follow-up. A Kaplan-Meier analysis plot for the probability to achieve pregnancy per month related to total follow-up time is presented in Figure 1.

**Table I:** Reproductive outcomes of patients with a dysmorphic uterus whom underwent hysteroscopic metroplasty

Dysmorphic uterus	Patients (n)	Mean age (years)	Pregnancies (n, %)	Live births (n, %)	Abortion (n, %)
Total “T” Shaped	190	36.8	152 (80.0)	147 (77.3)	17 (11.1)

**Figure 3:**
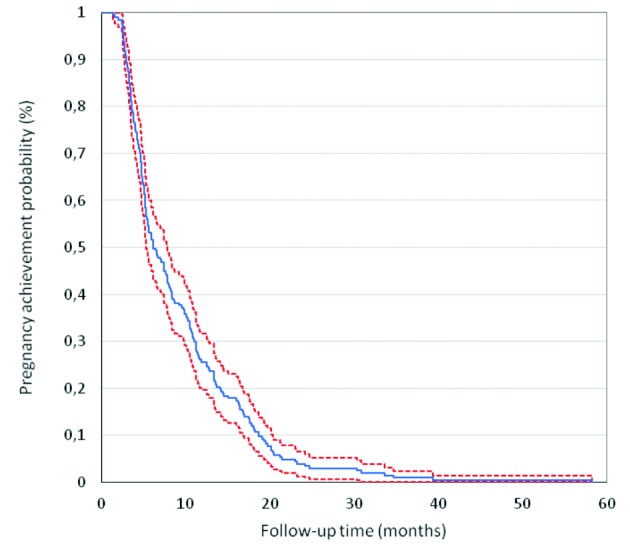
Kaplan-Meier life table analysis plot for pregnancies achieved per month.

No ectopic pregnancies or stillbirths were recorded. Thirty-four out of 152 pregnant women (21 twin and 13 singleton pregnancies) resulted in preterm delivery (34/152, 22.3%), and out of all gestations, 37 (24.3%) were twin pregnancies. Eighty-six patients (56.5%) were delivered by cesarean section. The rest had normal vaginal delivery.

Second-look hysteroscopy was performed in 93.7% of the patients. Ninety-one out of 190 (47.8%) women had a previous gestation, with only 1 born baby.

The mean time between metroplasty and diagnosis of pregnancy was 8.7 months (1.54 – 39.3 months), with the earliest pregnancy being diagnosed at 1.4 months. Out of the 152 total pregnancies, 146 were achieved employing assisted reproductive technologies. From theses, 79 (51.9%) were part of the oocyte donation program (fresh and freeze- thaw cycles). The other six patients accomplished pregnancy spontaneously (mean time to pregnancy after surgery 4.5 months). Ninety-six percent of gestations were achieved between 1 – 3 IVF cycles. Furthermore, 92% of the live births were born between 1 to 3 IVF cycles. With the practice of just 1 IVF cycle, 88 (77.8%) pregnancies were obtained, with a mean time from surgery to gravidness of 6.5 months. Additionally, 86 (76.1%) of the live births were from this subgroup. The data and reproductive outcomes corresponding to IVF cycles 1 to 3 are presented in Table II.

**Table II:** Reproductive outcome after metroplasty with the use of 1 – 3 IVF cycles.

IVF cycles after metroplasty	Patients (n)	Pregnancies (n, %)	Live births (n, %)	Mean time to pregnancy (months)
1	113	88 (77.8)	86 (76.1)	6.5
2	48	38 (79.1)	36 (75.0)	11.6
3	16	15 (93.7)	14 (87.5)	14.2
Total (1-3)	177	141 (79.6)	136 (76.8)	6.56

## Discussion

Congenital uterine anomalies have been implicated in women diagnosed with recurrent miscarriage and infertility (Serensen, 1988; Kupesic, 2001). According to the literature, although significant variation exists, the prevalence of congenital Müllerian anomalies in the infertile population is around 7.3% and 16.7% in those with recurrent miscarriage (Saravelos et al., 2008)

It is not clear whether there is a reasonable biological mechanism to link the presence of congenital Müllerian anomalies and diminished reproductive outcomes. Many attempts have been made to explain this phenomenon including the study of implantation failure and recurrent abortion. However, no clear evidence has been yet identified (Chan et al., 2011; Fernandez et al., 2011). The etiology of reproductive failure in these women remains unclear. The mechanisms by which dysmorphic uteri cause infertility and early pregnancy loss have not been established. However, a recent systematic review and meta-analysis found the presence of congenital uterine anomalies to be associated with a reduced probability of pregnancy (equally for natural and ART cycles). Still, this conclusion only reached statistical significance when summing up both groups (Fernandez et al., 2011). In their systematic review, Chan and colleagues (2011) reported diminished fertility outcomes, increased rates of miscarriage and augmented preterm delivery rates, in patients with dysmorphic uteri (canalization defects), and arcuate uterus (superior third septum) was found explicitly associated with second-trimester miscarriage.

Today we understand that a “T” shaped uterus can also have a primary or an acquired origin (adhesions) ( Fernandez et al., 2011). Historically, a “T” shaped uterus has been related to a congenital malformation (DES exposure), and infertility has been reported to be more common in dysmorphic uteri compared to a normal uterine cavity. Thus, the results presented in this study are promising in regard of the reproductive outcomes, and are consistent with prior evidence from other revisions of hysteroscopic metroplasty in the dysmorphic uterus (Nagel and Malo, 1993; Katz et al., 1996; Garbin et al., 1998; Fernandez et al., 2011).This study comprises a greater number of patients than those previously reported in the literature. Interestingly, and as opposed to many precedent publications, the present study shows how higher pregnancy and live birth rates are related to a first IVF cycle post-surgery. These values are 77.8% and 76.1%, respectively. The mean time to pregnancy was 6.5 months. Likewise, the Kaplan-Meier life table analysis plot for pregnancies achieved per month showed how 86% gravidities were obtained with between 1 to 3 IVF cycles, with an associated live birth rate of 76.8% and a mean time from metroplasty to surgery of 6.5 months. Although this is an observational study and no definitive conclusions could be drawn with this evidence, it shows a substantial relation between the metroplasty for dysmorphic uteri and promising reproductive outcomes (pregnancy and live birth rates). Furthermore, our findings are in line early studies published, on both, dysmorphic and in septate uteri, that presented successful outcomes (Nagel and Malo, 1993). Fedele et al. (1993) showed a cumulative pregnancy and birth rates, at 36 months, between 89% and 75%, with 80% in the septate uterus group and 67% in the subseptate uterus group. Other authors have published similar results (Katz et al., 1996; Fernandez et al., 2011).

Hysteroscopic metroplasty was found to be a safe procedure. No intraoperative or postoperative complications and no associated morbidity were reported. Due to the deficiency of prospective, randomized, controlled trials, there is lack of consensus on whether infertility is an indication for metroplasty. However, this procedure has presented satisfactory results in pregnancy and live-birth rates at a global scale. Second-look hysteroscopy was performed in 93.7% of patients, and it was proved safe, demonstrating that metroplasty leds to good anatomical results, with the possibility to detect and treat possible adherences, as published by other authors (Fernandez et al., 2011; Ferro and Montoya, 2016).

Nevertheless, this actual data cannot demonstrate a causal-effect relationship between dysmorphic uteri and infertility. Counting on its low morbidity and complication rate, plus, its successful practice, metroplasty should be considered to treat women with recurrent implantation failure due to dysmorphic uteri. There is dire need for a prospective, randomized, controlled study to support this evidence.

## Conclusions

This study and our experience at the Instituto Valenciano de Infertilidad (IVI) put forward hysteroscopic metroplasty as a treatment to improve pregnancy and live birth rates in women diagnosed with a dysmorphic uterus, in particular those with a T-shaped uterus and history of recurrent implantation failure In line with the existent literature, the application of this procedure represents a safe method with minimal complication and morbidity rates. Although this is an observational study and no definitive conclusions can be drawn from this evidence, this publication illustrates a positive relation between hysteroscopic metroplasty for dysmorphic uteri and satisfactory reproductive outcomes [pregnancy and live birth rates]. However, prospective, randomized, controlled studies are needed to support these results.
